# Crystallinity Tuning of Na_3_V_2_(PO_4_)_3_: Unlocking Sodium Storage Capacity and Inducing Pseudocapacitance Behavior

**DOI:** 10.1002/advs.202203552

**Published:** 2022-12-11

**Authors:** Hongyang Ma, Bangchuan Zhao, Jin Bai, Peiyao Wang, Wanyun Li, Yunjie Mao, Xiaoguang Zhu, Zhigao Sheng, Xuebin Zhu, Yuping Sun

**Affiliations:** ^1^ Key Laboratory of Materials Physics Institute of Solid State Physics HFIPS Chinese Academy of Sciences Hefei 230031 P. R. China; ^2^ Science Island Branch University of Science and Technology of China Hefei 230026 P. R. China; ^3^ High Magnetic Field Laboratory HFIPS Chinese Academy of Sciences Hefei 230031 P. R. China

**Keywords:** disorder, M1 sites, Na_3_V_2_(PO_4_)_3_, pseudocapacitance, sodium‐ion batteries

## Abstract

As a promising cathode material of sodium‐ion batteries, Na_3_V_2_(PO_4_)_3_ (NVP) has attracted extensive attention in recent years due to its high stability and fast Na^+^ ion diffusion. However, the reversible capacity based on the two‐electron reaction mechanism is not satisfactory limited by the inactive M1 lattice sites during the insertion/extraction process. Herein, self‐supporting 3D porous NVP materials with different crystallinity are fabricated on carbon foam substrates by a facile electrostatic spray deposition method. The V^5+^/V^4+^ redox couple is effectively activated and the three‐electron reactions are realized in NVP for sodium storage by a proper crystallinity tuning. In a disordered NVP sample, an ultra‐high specific capacity of 179.6 mAh g^−1^ at 0.2 C is achieved due to the coexistence of redox reactions of the V^4+^/V^3+^ and V^5+^/V^4+^ couples. Moreover, a pseudocapacitive charge storage mechanism induced by the disordered structure is first observed in the NVP electrode. An innovative model is given to understand the disorder‐induced‐pseudocapacitance phenomenon in this polyanion cathode material.

## Graphical abstract

The V^5+^/V^4+^ redox couple in Na_3_V_2_(PO_4_)_3_ (NVP) can be activated through a proper crystal phase controlling method based on the characteristics of NVP. The obtained NVP material has an ultra‐high specific capacity of 179.6 mAh g^−1^. In addition, the disordered structure in NVP can induce pseudocapacitance behavior, which is caused by the reduced interaction force between the inserted ions.

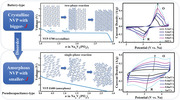

## 1 Introduction

In the past few decades, lithium‐ion batteries (LIBs) have been widely used as rechargeable energy storage devices. However, the scarcity of lithium resources severely limited the development of these energy storage systems. The low cost and abundant resources of sodium make sodium‐ion batteries (SIBs) a potential alternative to LIBs, especially in applications of large‐scale energy storage occasion.^[^ ^1^ ^]^ Although SIBs have a similar working principle with LIBs, the solid‐state diffusion kinetics of Na^+^ ion is sluggish owing to the larger radius of Na^+^ ion than that of Li^+^ (0.98 Å vs 0.69 Å). Selecting a proper electrode material is especially important in designing high‐performance SIBs.^[^ ^2^ ^]^ The sodium superionic conductor (NASICON) material always has an open 3D structure, which is suitable for the rapid migration of sodium ions and may be a potential electrode material for Na^+^ storage.^[^ ^3^ ^]^ Among the series of NASICON structural materials, Na_3_V_2_(PO_4_)_3_ (NVP) has drawn intensive attention due to its stable structure, large capacity, and high working potential.^[^ ^4^ ^]^

In the rhombohedral NASICON structure NVP, sodium ions occupy two inequivalent Wyckoff sites, 1/3 located at 6b sites (M1) and 2/3 located at 18e sites (M2) with sixfold and eightfold oxygen coordination, respectively.^[^ ^5^ ^]^ Na^+^ ions may diffuse through M1‐M2‐M1 zigzag or M2‐M2 direct jump pathway in the material.^[^ ^6^ ^]^ Previous studies turned out that M1 sites in NVP are inactive during charge–discharge processes. Na^+^ ion migrates in the material through the M1‐M2 path, which will increase the average Na—O distance. The migration is a strong endothermic process, which is difficult to achieve thermal equilibrium. Moreover, the migration process is also energetically unfavorable with a relatively high activation barrier from the kinetics point.^[^ ^6^, ^7^ ^]^ Hence, the reversible sodium (de)intercalation in NVP only occurred at M2 sites.^[^ ^8^ ^]^ It means that only two Na^+^ ions per formula could participate in the electrochemical reaction in Na_3_V_2_(PO_4_)_3_ via V^4+^/V^3+^ redox couple (3.4 V vs Na^+^/Na), delivering a theoretical specific capacity of 117.6 mAh g^−1^ and yielding an energy density of 396 Wh kg^−1^ for sodium storage.^[^ ^9^ ^]^ To elevate the specific capacity of Na_3_V_2_(PO_4_)_3_, triggering M1 sites and then realizing the V^5+^/V^4+^ redox couple is a possible and effective strategy. Lalère et al.^[^ ^10^ ^]^ successfully realized the V^5+^/V^4+^ redox couple in NVP through a proper substitution of aluminum for vanadium. However, the valence of the substituted Al^3+^ cannot change during this electrochemical window, the specific capacity of the material was not improved. Hu et al.^[^ ^6^ ^]^ found that Zn^2+^ could be mixed‐occupied at both the M1 and M2 sites in Na_3_V_2_(PO_4_)_3_ when worked as cathode material for aqueous Zn ion batteries. Such a mixed occupation could activate the M1 site for Zn^2+^ (de)intercalation in NVP, leading to the concerted migration of Na^+^/Zn^2+^ ions between M1 and M2 sites. Similar problem of blocked ionic pathways also exists in NaFePO_4_.^[^ ^11^, ^12^ ^]^ Xiong et al.^[^ ^12^ ^]^ tried to improve the electrochemical performance of a sodium storage inactive material (the maricite phase NaFePO_4_) through the disorder degree tuning by a mechanochemical route. The derived NaFePO_4_ cathodes containing both amorphous and maricite phases exhibit much improved sodium storage performance with an initial capacity of 115 mA h g^−1^ at 1 C and an excellent cycling stability.

Herein, self‐supporting 3D porous NVP material was prepared by electrostatic spray deposition (ESD) method on a melamine sponge‐derived carbon foam (CF) substrate. The resultant NVP materials have a disordered structure with coexistence of nanocrystalline and amorphous phases, which shows quite different thermodynamic and kinetic behaviors to the well‐crystalline NVP sample. When used as binder‐free cathode of SIBs, the disordered structure can endow the V^5+^/V^4+^ couple activated and the third Na^+^ ion can participate in the redox procedure, bringing a three‐electron reaction process and excellent electrochemical performance. In addition, an exotic disorder‐induced pseudocapacitance behavior was first observed in NVP material.

## 2 Results and Discussion

**Figure 1:**
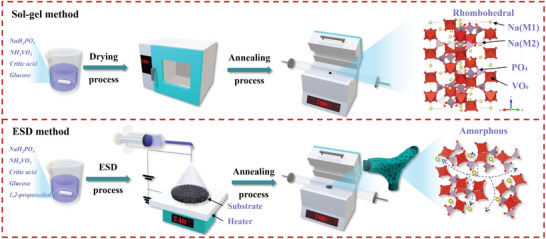
Schematic illustration of the synthesis processes of Na_3_V_2_(PO_4_)_3_ by sol–gel and ESD methods.

**Figure 2:**
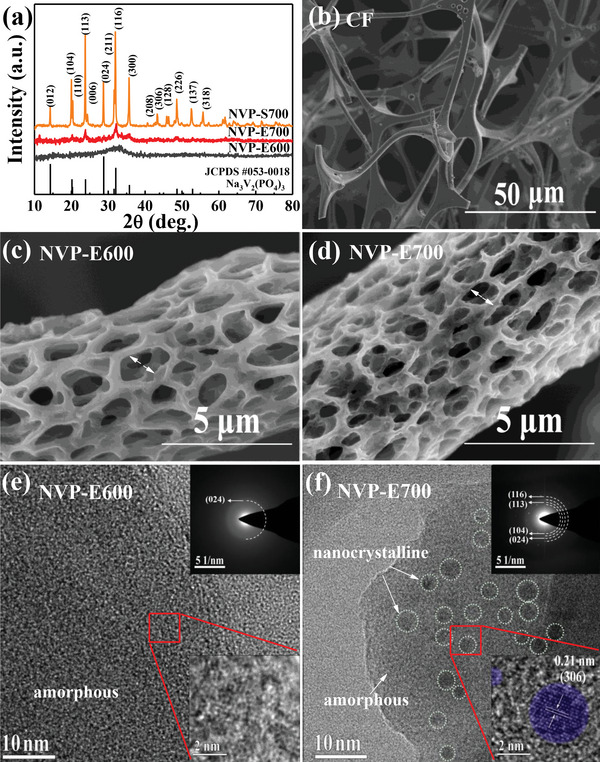
a) XRD patterns of the NVP‐S700, NVP‐E700, and NVP‐E600 samples. FE‐SEM images of b) carbon foam, c) NVP‐E600, and d) NVP‐E700. HRTEM images of e) NVP‐E600 and f) NVP‐E700, the insets in (e, f) show the SAED patterns of NVP‐E600 and NVP‐E700.

Self‐supporting 3D porous NVP films were prepared on the CF substrate by ESD method. NVP‐E700 and NVP‐E600 were obtained by adjusting the sintering temperature at 700 and 600 °C, respectively. For comparison, NVP‐S700 sample was synthesized by a conventional sol–gel method. The synthesis procedure of the materials is schematically shown in **Figure** **1**. To investigate the crystalline property of the NVP samples prepared by ESD and sol–gel methods, X‐ray diffraction (XRD) data were recorded and are presented in **Figure** **2**a. The NVP‐S700 material prepared by the sol–gel method has a typical rhombohedral NASICON structure with *R‐3c* space group (JCPDS no. 053‐0018). The 700 °C annealed ESD sample NVP‐E700 has the similar rhombohedral NASICON structure with that of NVP‐S700, but only a few main characteristic peaks presented in the XRD pattern. Compared to the NVP‐S700 sample, the peak intensity of NVP‐E700 sample becomes weaker and the peak shape becomes obviously broadened. The average grain size of the NVP‐E700 sample calculated using the Scherrer equation is around 4 nm, indicating the nanocrystalline feature of the NVP‐E700 sample. As the annealing temperature drops, the peak in the XRD pattern will be further broadened even disappeared. There is only one very broaden peak at about 32° in the XRD pattern of the NVP‐E600 sample, consistent with the (116) atomic planes of NVP, as shown in Figure 2a. The result shows that the NVP‐E600 sample may have an amorphous nature as discussed below.

Field‐emission scanning electron microscopy (FE‐SEM) measurements were employed to investigate the morphology of the CF substrate and the NVP film supported by CF. Figure 2b shows the morphology of CF, which was prepared by carbonizing the melamine sponge under argon atmosphere. The CF material has a porous structure composed of smooth surface skeleton, and the diameter of the skeleton is about 5 µm. The morphology and microstructure of the NVP‐E600 and NVP‐E700 samples are shown in Figure 2c,d, respectively. The NVP materials are uniformly deposited on the surface of CF for both the two samples, showing a 3D porous structure with pore diameter ranging from 1 to 2 µm. The formation of the porous structure is related to the polycondensation and decomposition processes of the 1–2 propanediol solvent induced by the high temperature substrate.^[^ ^13^ ^]^ It can be seen from the cross‐section that the thickness of the deposited film is about 1 µm (Supporting Information, Figure S1b). Although the two samples obtained at two different annealing temperatures have similar morphology, their surface roughness is quite different. The surface shrinkage of the sample annealed at higher temperature (NVP‐E700) is more obvious. The composition of the NVP‐E700 film is further confirmed by energy‐dispersive X‐ray analysis method. The elements Na, V, and P coexisted and uniformly distributed on the surface of CF, and the normalized atomic ratio is consistent with the stoichiometric ratio of Na_3_V_2_(PO_4_)_3_ for the NVP‐E700 sample (Supporting Information, Figure S2). The carbon contents in the NVP‐S700, NVP‐E700, and NVP‐E600 samples were determined by thermogravimetric measurements, which are 4.4, 4.7, and 4.6 wt%, respectively (Supporting Information, Figure S3). Inductively coupled plasma optical emission spectra measurement further confirmed that the molar ratio of Na:V:P in all samples is 3:2:3 (Supporting Information, Table S1).

Although FE‐SEM characterization can provide some important surface morphology information, the bulk structure of the material cannot be thoroughly reflected. To further ascertain the local structure of the two materials prepared by the ESD method, high‐resolution transmission electron microscopy (HRTEM) measurement was employed and the results are shown in Figure 2e,f. No well‐defined lattice fringes existed in the HRTEM image of the NVP‐E600 sample (Figure 2e). Moreover, the selected‐area electron diffraction (SAED) image only shows some fuzzy halos without any diffraction spots, revealing the amorphous structural property of the material. The result is also consistent with the fact that no obvious diffraction peak exists in the XRD pattern. However, a large number of dark spots with the size of 2–4 nm exist in the HRTEM bright field image of the NVP‐E700 sample (Figure 2f), the contrast of these dark spots is caused by the diffraction of the NVP nanocrystalline. The amorphous phase NVP materials wrap around the nanocrystalline. As amplifying the dark spots, well‐defined lattice fringes can be observed, which correspond to the (306) crystal plane with spacing distance of 0.21 nm for the NVP material. From the SAED image (inset of Figure 2f), it can be noted that numerous discrete light spots distributed around the fuzzy halos, which is induced by the diffraction of NVP nanocrystalline, indicating a certain degree of crystallinity occurred in the material.

**Figure 3:**
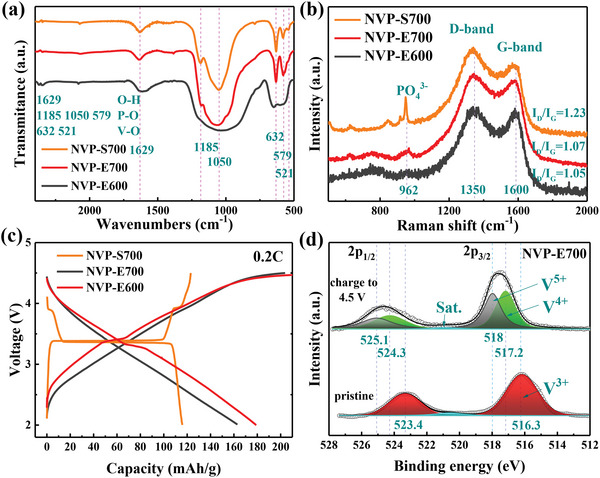
a) FT‐IR spectra and b) Raman spectra of NVP‐S700, NVP‐E700, and NVP‐E600 samples. c) The first cycle galvanostatic charge–discharge curves of the as‐prepared cathodes at 0.2 C. d) High‐resolution XPS spectra of V (2p) for the NVP‐E700 sample before and after charged to 4.5 V.

Fourier transform infrared (FT‐IR) spectra were recorded to further investigate the composition of the samples as shown in **Figure** **3**a. Several characteristic peaks can be detected in the FT‐IR spectra, which are typical representative of the chemical bonds in the NASICON‐type structure: the peaks at 1185, 1050, 579 cm^−1^ are relative to the vibration of P—O bonds in PO_4_ tetrahedra and the peaks located at 632 and 521 cm^−1^ are attributed to the vibration of V—O bonds in VO_6_ octahedra.^[^ ^14^ ^]^ It is worth noting that the peak width gradually increases with the decreasing crystallinity from NVP‐S700, NVP‐E700 to NVP‐E600, indicating the absorbing energy of the vibration will be changed. For the NVP‐S700 sample, a well‐crystallized material, the lengths and angles of the P—O and V—O bonds are single and the FT‐IR peaks are much sharp. As to the NVP‐E700 sample, the atoms of amorphous and the surface atoms of nanocrystalline account for the majority, which causes the distortion of the PO_4_ tetrahedra and VO_6_ octahedra in the NASICON‐type structure, perturbing their vibration frequencies. For the amorphous NVP‐E600, its disordered structure causes a larger range of vibration frequency, so the fine structure of FT‐IR spectra is almost lost.

Raman spectroscopy was tested to further investigate the structure of the studied NVP samples (Figure 3b). Two obvious peaks were observed in the Raman spectroscopy of all three samples at 1350 and 1600 cm^−1^, which are typical characteristics of carbon materials, corresponding to D band (disordered carbon sp^3^‐coordinated behavior) and G band (graphene carbon sp^2^‐coordinated behavior).^[^ ^15^ ^]^ The relative intensity ratio between the D and G bands (*I*_D_/*I*_G_) is commonly utilized to reflect the degree of the graphitization in the material. The calculated *I*_D_/*I*_G_ of NVP‐S700, NVP‐E700, and NVP‐E600 are 1.23, 1.07, and 1.05, respectively. Due to the CF substrate, the sample prepared by ESD method exhibits a high degree of graphitization. In the Raman spectroscopy of the NVP‐S700 sample, there also exists an obvious peak near 962 cm^−1^, which is induced by the tensile vibration mode of PO_4_ in Na_3_V_2_(PO_4_)_3_.^[^ ^16^ ^]^ Such a characteristic peak becomes weaker and broader in the Raman spectroscopy of the NVP‐E700 and NVP‐E600 samples, indicating that the degree of the crystallinity is weak in the Na_3_V_2_(PO_4_)_3_ materials prepared by ESD method, which is consistent with that of the above XRD and FT‐IR tests.

Based on the analysis results of XRD, SEM, TEM, FT‐IR, and Raman spectroscopy above, an amorphous or nanocrystalline state material was formed by ESD method and subsequent high‐temperature annealing process, which has also been presented in previous reports.^[^ ^17^ ^]^ In fact, the formation of amorphous or nanocrystalline state may be contradictory to the high‐temperature annealing process, since the amorphous or nanocrystalline particles with larger surface energy should easily grow up to reduce their energy at high temperatures.^[^ ^18^ ^]^ Here, we propose a possible model to explain the formation mechanism of such an amorphous or nanocrystalline NVP phase material (Supporting Information, Figure S4). In the ESD process, the electric field force and friction can overcome the surface tension, the precursor solution will break into charged droplets and deposited on the heated substrate at 240 °C. The rapidly heated process making the droplets evaporated instantaneously and the precursor solution concentrated and supersaturated quickly. Simultaneously, the solute atoms precipitated and a large number of nanocrystal nucleus formed with a very high nucleation rate. In such an NVP precursor formation process, the hydrogen and hydroxyl in the organic components (citrate, glucose) of the solution will combine at 240 °C and separate out in the form of structural water, and C=O and C=C bonds could be formed in the material, accompanying the occurrence of a certain degree cleavage and many micropores inside the material. In following annealing process, these organic precursors will be carbonized. EDS mapping results verify that the element C uniformly distributed in the NVP‐E700 (Supporting Information, Figure S2). The formed carbon source and micropores could effectively pin up the dislocations, hindering the movement of grain boundaries and weakening the agglomeration and growth of NVP grains during the annealing process.^[^ ^19^ ^]^ That is to say, the nucleation and growth of NVP phase are confined in a very small area, resulting in the amorphous or nanocrystalline morphology.

To ascertain the effect of crystallinity on the sodium storage performance of Na_3_V_2_(PO_4_)_3_ materials, half‐coin cells were assembled by using the as‐synthesized NVP‐S700, NVP‐E700, and NVP‐E600 as the working cathode and sodium metal as the counter electrode. Figure 3c depicts the first cycle galvanostatic charge–discharge curves of the three cells at 0.2 C rate. The electrode materials with different crystallinity show quite different charge–discharge curves. First, the NVP‐E700 sample shows the highest initial reversible capacity of 179.6 mAh g^−1^, far beyond than the theoretical capacity value with 2 sodium ions insertion/extraction. The initial capacity of NVP‐E700 is 155% of the NVP‐S700 sample (115.9 mAh g^−1^) and 109.3% of the NVP‐E600 sample (163.7 mAh g^−1^). Second, there exists a long voltage plateau at 3.4 V in the charge–discharge curve of the NVP‐S700 sample, corresponding to the V^4+^/V^3+^ redox reaction.^[^ ^20^ ^]^ Such a plateau is diminished obviously for the NVP‐E700 sample, and the charge–discharge curve shows a rather sloping line in the whole potential range. For the NVP‐E600 sample, the plateau almost disappeared and the charge–discharge curve became an inclined profile. In order to eliminate the influence of the CF on electrochemical properties of the NVP‐E700 and NVP‐E600 samples, the charge–discharge curve of the CF substrate was also measured (Supporting Information, Figure S5). The result shows that the capacity contribution of CF substrate is almost negligible.

To interpret the origin of the much higher initial capacity of NVP‐E700, X‐ray photoelectron spectroscopy (XPS) was measured for the electrode before and after charging it to 4.5 V. The wide‐range spectrum (Supporting Information, Figure S6a) presents the existence of Na, V, P, O, N, and C. The measured results along with its fitting curves are displayed in Figure 3d. The scale of the binding energy (BE) is calibrated by setting the BE of C 1s to 284.5 eV. The V 2p core level spectra of the pristine NVP‐E700 electrode exhibit two peaks at 523.4 and 516.3 eV, which match well with V 2p_1/2_ and V 2p_3/2_ of V^3+^ ions, respectively.^[^ ^21^ ^]^ After charging the electrode to 4.5 V, the spectrum can be fitted to two sets of peaks with equal areas. The two peaks at 524.3 and 517.2 eV match well with V 2p_1/2_ and V 2p_3/2_ of V^4+^ ions, and the other two peaks are related to V^5+^ 2p_1/2_ (525.1 eV) and V^5+^ 2p_3/2_ (518.0 eV), indicating that three sodium ions per formula could be extracted from the sample.^[^ ^22^ ^]^ As a comparison, we also did XPS test on the NVP‐S700 sample (Supporting Information, Figure S6b). After charging to 4.5 V, only V 2p_1/2_ (524.2 eV) and V 2p_3/2_ (517.1 eV) XPS peaks related to V^4+^ existed in the spectra, meaning that only two sodium ions per formula could be extracted in this voltage range.

**Figure 4:**
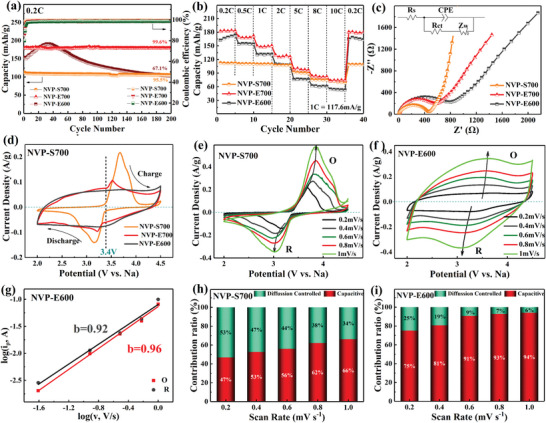
a) Cycle performance, b) rate performance, c) electrochemical impedance spectra, and d) CV curves of the NVP‐S700, NVP‐E700, and NVP‐E600 electrodes, the CV curves of e) NVP‐S700 electrode and f) NVP‐E600 electrode at different scan rates from 0.2 to 1 mV s^−1^. g) The log (*i*_p_) versus log (*v*) curves of NVP‐E600 electrode. The contribution ratios of pseudocapacitive for h) NVP‐S700 and i) NVP‐E600 electrodes at each scan rate.

**Figure** **4**a shows the cycle performance of the NVP‐S700, NVP‐E700, and NVP‐E600 electrodes at 0.2 C (1 C = 117.6 mA g^−1^). The well‐crystallined NVP‐S700 electrode delivered an initial specific capacity of 115.9 mAh g^−1^ and the capacity remains 110.7 mAh g^−1^ after 200 cycles, corresponding to a capacity retention of 95.5%. Relative to NVP‐S700, the NVP‐E700 electrode delivered a much higher initial discharge capacity of 179.6 mAh g^−1^ and the capacity attenuation is negligible even after 200 cycles with capacity retention of 99.6%, showing an outstanding cycle stability property. The amorphous NVP‐E600 sample also delivers a high initial capacity of 165 mAh g^−1^ and the capacity increases to 190 mAh g^−1^ in the first 30 cycles, and then decreases sharply in the following cycles. The NVP‐E600 and NVP‐E700 electrodes show lower Coulombic efficiency than the NVP‐S700 electrode that is caused of defects in the amorphous structure, which will lead to side reactions during charge/discharge process and result in irreversible losses of sodium. Figure S8 in the Supporting Information shows the SEM, TEM, and XPS spectra of V (2p) of NVP‐E600 and NVP‐E700 electrodes after cycling. It can be seen from the SEM image that the surface of NVP‐E600 is rougher after cycling, and the morphology of NVP‐E700 was maintained. HRTEM images show that the NVP‐E600 sample is still amorphous after the cycle, the NVP‐E700 sample retains the nanocrystalline. XPS spectra show that a small amount of V in NVP‐E600 and NVP‐E700 samples is reduced to bivalent after cycling, indicating that V^3+^/V^2+^ redox couple also partially participates in the reaction. The excellent performance of NVP‐E700 electrode could be mainly determined by the exotic structure of coexistence of nanocrystalline and amorphous phases as discussed above. More spaces in the amorphous structure can reduce the volume change during the cycle and stable bonding in crystalline phase can keep the structure from collapsing.^[^ ^23^ ^]^ For an absolute amorphous NVP‐E600 electrode, the sodium storage sites could be exposed in the first few cycles and then the capacity increased.^[^ ^24^ ^]^ However, due to the lack of stable support, the structure may collapse in the subsequent cycles, resulting in the capacity decrease.

The rate performance of the NVP‐S700, NVP‐E700, and NVP‐E600 samples was tested at current densities of 0.2 C to 10 C as shown in Figure 4b. The NVP‐E600 and NVP‐E700 samples showed much higher capacity at lower current rates than that of the NVP‐S700 electrode, especially the NVP‐E700 electrode. The extra capacity could be originated from the activation of V^5+^/V^4+^ redox couple and the participation of the third sodium ion as discussed before. The NVP‐E700 electrode delivers charge capacities of 179.6, 169.3, 149.2, 129.5, 98.2, 82.8, and 73.5 mAh g^−1^ at corresponding C‐rates of 0.2 C, 0.5 C, 1 C, 2 C, 5 C, 8 C, and 10 C. When the current density is restored from 10 C to 0.2 C, the reversible capacity recovers to 179.1 mAh g^−1^, which is almost the same with its initial value, demonstrating an excellent reversibility property. Although the NVP‐E600 electrode also delivered a high capacity of 165 mAh g^−1^ at low current density of 0.2 C, the capacity decreased fast to 76.1 mAh g^−1^ when the current density is higher than 5 C, which is 77.5% of NVP‐E700 at the same rate, even lower than NVP‐S700. The rate properties were mainly related to the disorder structure of the materials: on the one hand, the amorphous phase in NVP could weaken the bondage of sodium ions by oxygen, realizing three‐electron transfer in NVP and much higher capacity at low current density; on the other hand, the electron transportation is hindered due to the scattering effect of the disorder structure.^[^ ^25^ ^]^ Excessive disorder structure will adversely affect the rate performance of the material.

To get direct evidence of the influence of amorphous structure on the electrochemical reaction dynamics for the NVP samples, the electrochemical impedance spectra (EIS) measurements were tested (Figure 4c). All the three EIS curves show a semicircle at high frequencies standing for charge‐transfer resistance (*R*_ct_) and a straight line in the low‐frequency region reflecting the Warburg resistance (*Z*_W_), which is associated with Na^+^ ion diffusion process in NVP. It is found that the semicircle is larger in the NVP‐E600 sample than that in NVP‐E700 and NVP‐S700, indicating a higher charge‐transfer resistance. The EIS plots were fitted based on the equivalent circuit shown in the inset of Figure 4c. In this circuit, *R*_s_ stands for the bulk resistance and constant phase element (CPE) is related to the double layer capacitance. The fitting results are listed in Table S2 in the Supporting Information. The *R*_ct_ of the NVP‐S700, NVP‐E700, and NVP‐E600 sample are 428, 712, and 827 Ω, respectively. Meanwhile, the diffusion coefficients of sodium ions (*D*_Na_^+^) of all samples are calculated based on the Warburg impedances in the low‐frequency range of the EIS spectra. The equations can be expressed as

(1) $$Z^{\prime }=R_{s}+R_{\mathrm{ct}}+\sigma \omega^{-1/2}$$

(2) $$D_{{\mathrm{Na}}^{+}}=\frac{R^{2}T^{2}}{2A^{2}n^{4}F^{4}C^{2}\sigma^{2}}$$

where *σ* denotes the Warburg factor, *R* denotes the gas constant, *T* is the absolute temperature, *A* is the surface area of the cathode, *n* is the number of electrons per molecule during oxidization, *F* is the Faraday constant, and *C* reflects the concentration of sodium ions (3.47 × 10^−3^ mol cm^−3^). The relationship between *Z'* and *ω* ^−1/2^ used for the calculations is displayed in Figure S7a in the Supporting Information. The calculated *D*_Na+_ of NVP‐E600 is 1.05 × 10^−15^ cm^2^ s^−1^, which is lower than that of NVP‐E700 (2.01 × 10^−15^ cm^2^ s^−1^) and NVP‐S700 (9.77 × 10^−14^ cm^2^ s^−1^), which is consistent with the rate performance as discussed above. The result shows that the rate performance and capacity of NVP materials can be well tuned by the crystalline state.

To understand the electrochemical reaction mechanism in the charging and discharging processes, the cyclic voltammetry (CV) curve of the three samples was tested in the voltage range of 2–4.5 V with a scan rate of 0.2 mV s^−1^ (Figure 4d). It can be seen that the well‐crystallined NVP‐S700 sample has a pair of obvious redox peaks located at 3.6 and 3.2 V positions in the CV curve, which indicates that the electrode reaction is concentrated in this voltage range. Although the CV curve of the NVP‐E700 sample also shows a pair of redox peaks, the peak is much weaker than that of NVP‐S700. For the amorphous NVP‐E600 sample, the redox peak almost disappeared and CV curve tends to be rectangular. The phenomenon shows that the charge‐storage mechanism of the NVP electrodes was changed by tuning crystallinity.

The CV curves at different scan rates were further analyzed to understand the charge‐storage mechanism of the samples. Figure 4e,f and Figure S7c in the Supporting Information exhibit the CV curves of the NVP‐S700, NVP‐E700, and NVP‐E600 electrodes at different scan rates from 0.2 to 1 mV s^−1^. The peak intensity of both the CV curves increases with the increasing scan rates. Figure S7e,f in the Supporting Information and Figure 4g present the log (*i*_p_) versus log (*v*) plots of the samples for each redox peak. All the plots show nearly straight line and the parameter *b* can be obtained by fitting the line. For NVP‐S700, the calculated *b* values of the reduction (R) and oxidation (O) peaks are 0.54 and 0.58, respectively (Supporting Information, Figure S7e), which indicates that the sodiation/desodiation reactions in NVP‐S700 are mainly controlled by diffusion process, showing a battery‐type charge‐storage behavior.^[^ ^26^, ^27^ ^]^ While the *b* values exceeded 0.8 for NVP‐E700 (Supporting Information, Figure S7f) and 0.9 for NVP‐E700 (Figure 4g), means the sodiation/desodiation reactions are mainly controlled by a pseudocapacitance storage behavior.^[^ ^28^ ^]^ That is to say, in an NVP electrode, the charge‐storage mechanism changed from battery‐type to pseudocapacitance‐type as the material structure varied from crystalline to amorphous state. The pseudocapacitance contribution ratios to the whole capacity of NVP‐S700 and NVP‐E600 at different scan rates were calculated as shown in Figure 4h,i. The pseudocapacitance contribution ratios of both the samples increase significantly with the increasing scan rates. The pseudocapacitance contribution ratio of NVP‐E600 reaches 94% at a scan rate of 1 mV s^−1^, which is much higher than the value 66% of NVP‐S700. The results demonstrate that by adjusting the crystallinity, the charge‐storage behavior in the NVP electrode material can be changed, which updates our understanding to the NVP material. Previous reports showed that some battery‐type materials could exhibit pseudocapacitance charge‐storage behavior when converted the material from bulk into nanoscale state: the voltage curve becomes linear dependence with the stored charge and the CV curve becomes rectangular, which is called extrinsic pseudocapacitance.^[^ ^26^, ^29^ ^]^ The result shows that both the nanocrystallization and amorphization processes can bring the NVP material showing pseudocapacitive charge‐storage behavior, which is quite different to that reported earlier. The reason of such an exotic phenomenon will be comprehensively analyzed in the following.

Based on the above analysis, we can conclude that the electrochemical performance of NVP material can be well tuned by their crystallinity optimization. NVP material with different crystallinity have different voltage curves, which can be well explained by the lattice‐gas model.^[^ ^30^ ^]^ The model is defined as a guest–host system, where the host is an ordered site network and each site can be empty or occupied by a guest ion. For most intercalation compounds, the interaction between the inserted ions in the lattice cannot be ignored. Assuming that each ion only interacts with its neighboring ions, the contribution of this effect to the chemical potential should be proportional to the ion filling fraction. The potential *E* is then given by

(3) $$E\;=E^{0}\;-\frac{RT}{F}\ln(\frac{x}{1-x})\;+J\;(x-0.5)$$

here, *E* ^0^ is the initial potential (V), *R* is the ideal gas constant (8.314 J mol^−1^ K^−1^), *T* is the absolute temperature (K), *F* is the Faraday constant (96 485 C mol^−1^), *x* is the filling fraction of the intercalated ions, and *J* is the interaction parameter between the adjacent ions. The relationship between *E*, *x*, and *J* is shown in Figure S9 in the Supporting Information. When the interaction between adjacent ions is weak (0 ≤ *J* < 4*RT*/*F*), the intercalation or deintercalation is a single‐phase process and the *E–x* curve is an inclined straight line. When the interaction between adjacent ions is strong (*J* > 4*RT*/*F*), the *E–x* curve has a maximum and a minimum and the intercalation or deintercalation process exhibits a two‐phase reaction. Therefore, for the intercalation compounds material, the voltage curves are mainly affected by the interaction of the inserted ions in the host site network.

**Figure 5:**
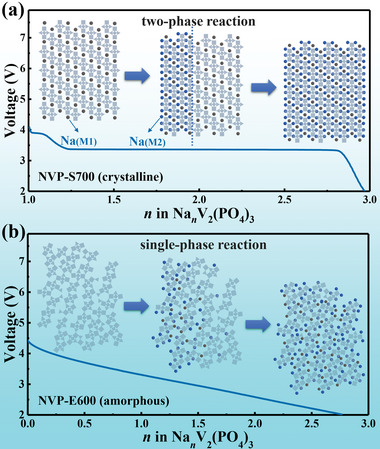
Schematic diagram of voltage curve and corresponding internal process of a) crystalline NVP and b) amorphous NVP, the small grown and purple circles represent Na atoms and the gray squares stand for the lattice.

Based on the lattice‐gas model, the voltage curves in Figure 3c were fitted by formula (3) and the fitted *J* values are 0.9, −1.9, and −2.1 for the NVP‐S700, NVP‐E700, and NVP‐E600 samples, respectively, as shown in Figure S10a–c in the Supporting Information. In the crystalline NVP‐S700 sample, the sodium ions are inserted in an ordered site network, which caused a large *J* value between sodium ions. According to the lattice‐gas model, there should exist a strong repulsive force between the successively inserted ions, making the insertion process be a local and ordered diffusion. Therefore, it is a typical NaV_2_(PO_4_)_3_‐Na_3_V_2_(PO_4_)_3_ phase transition process rather than random and uniform diffusion.^[^ ^16^, ^31^ ^]^ Based on the classic Gibbs phase rule, when the two phases with different compositions reach an equilibrium state, the chemical potential should be a constant and a voltage plateau in the discharge curve appeared, as shown in **Figure** **5**a.^[^ ^32^ ^]^ However, the long‐range order of lattice in the nanocrystalline NVP (surface disorder) and amorphous NVP (bulk disorder) sample is disrupted and the interaction between the inserted sodium ions is weakened, corresponding to a smaller *J* value. The inserted sodium ions can diffuse randomly and evenly in the electrode material, showing a tendency to a single‐phase reaction in the electrochemical process. Therefore, the voltage plateau in the discharge curve of such materials disappeared and the potential changes linearly with the increase of the sodium ion concentration, as shown in Figure 5b.^[^ ^32^ ^]^

In the charging process, the electrode reaction (Na_3_V_2_(PO_4_)_3_‐NaV_2_(PO_4_)_3_) in crystalline NVP‐S700 happens if the scan voltage (>3.4 V) makes the electric work higher than the Gibbs free energy change (Δ*G*) between the two phases. In discharge process, the Δ*G* makes the electrode reaction (NaV_2_(PO_4_)_3_‐Na_3_V_2_(PO_4_)_3_) happens when the scan voltage was under 3.4 V. Therefore, the electrode reaction current exhibits a pair of maximal values at a certain voltage in the CV curves. While for amorphous NVP, there is no phase transition in the whole voltage window. The disorder structure will diminish the variance of the site energy for the inserted ions, meaning that the diffusivity of the inserted ions is more independent of their concentration.^[^ ^33^ ^]^ The ions undergo semi‐infinite diffusion in the electrodes, and the diffusion rate (corresponding to the electrochemical reaction current) was just related to the voltage scan rate. Thus, the current tends to be constant in the entire voltage range, leading to the rectangular shape CV curve and showing the extrinsic pseudocapacitance behavior.

**Figure 6:**
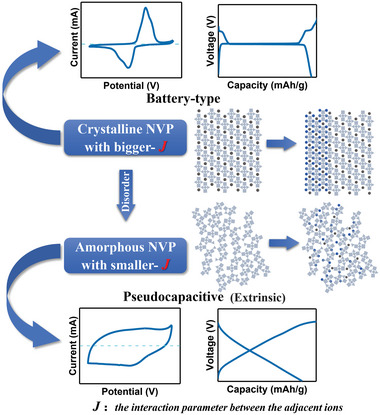
Schematic diagram of the relationship between *J* and pseudocapacitance charge‐storage behavior.

From the above analysis, the extrinsic pseudocapacitance in the NVP material is closely related to the *J* value (**Figure** **6**) which could be deduced, the extrinsic pseudocapacitance phenomenon is always appeared in an NVP system with lower *J* value. For crystalline NVP, the *J* value is large and it always exhibits battery‐type charge‐storage behavior. The *J* value can be reduced through preparing amorphous or nanoscale material. The inserted ions in such a disordered material could diffuse randomly and cause a disorder‐induced‐pseudocapacitance phenomenon. This pseudocapacitance behavior is another contributing factor to the outstanding sodium storage ability of the NVP‐E700 sample. For some battery‐type materials with two‐phase transition mechanism, due to the limiting of the ions diffusion at the phase interface, some regions cannot participate in the electrochemical reaction, resulting in partial capacity loss during the charge–discharge process.^[^ ^34^ ^]^ However, in a pseudocapacitive‐type material, the Na^+^ ion diffusion process will not be limited by such a two‐phase interface, and then the material shows an outstanding electrochemical performance, especially the capacity retention. Benefitting from the synergistic effect of the robust structure, the pseudocapacitive contribution, and the activation for V^5+^/V^4+^ redox couple, the NVP‐E700 material shows the best electrochemical performance in the three studied samples.

## 3 Conclusion

In summary, self‐supporting 3D porous NVP films were deposited on CF substrate by ESD method. The structural characterizations indicate that the NVP‐E700 film shows a certain degree of disordered structure. The disordered structure could weaken the bondage of oxygen to sodium ions, activating the V^5+^/V^4+^ redox couple, realizing three‐electron reaction process with a specific capacity of 179.6 mAh g^−1^ at 0.2 C. The disordered structure can also change the interaction between the inserted sodium ions in the host network site, leading to the rectangular‐like CV curve and the disappearance of plateau in the charge–discharge curve, behaving as a disorder‐induced‐pseudocapacitance. The pseudocapacitive contribution plays an important role in the charge storage of the NVP‐E700 sample. Our research demonstrates the importance of a disorder structure to the three‐electron reaction process and extrinsic pseudocapacitance in NVP cathodes for sodium‐ion batteries.

## Conflict of Interest

The authors declare no conflict of interest.

## Supporting information

Supporting Information

## Data Availability

The data that support the findings of this study are available from the corresponding author upon reasonable request.

## References

[1] a) W. Bi , G. Gao , G. Wu , M. Atif , M. S. AlSalhi , G. Cao , Energy Storage Mater. 2021, 40, 209;

[2] a) Y. Xu , Q. Wei , C. Xu , Q. Li , Q. An , P. Zhang , J. Sheng , L. Zhou , L. Mai , Adv. Energy Mater. 2016, 6, 1600389;

[3] Q. Ni , Y. Bai , F. Wu , C. Wu , Adv. Sci. 2017, 4, 1600275. 10.1002/advs.201600275 PMC5357992 28331782

[4] a) W.‐J. Li , C. Han , W. Wang , F. Gebert , S.‐L. Chou , H.‐K. Liu , X. Zhang , S.‐X. Dou , Adv. Energy Mater. 2017, 7, 1700274;

[5] Z. Jian , Y. S. Hu , X. Ji , W. Chen , Adv. Mater. 2017, 29, 1601925. 10.1002/adma.201601925 28220967

[6] P. Hu , Z. Zou , X. Sun , D. Wang , J. Ma , Q. Kong , D. Xiao , L. Gu , X. Zhou , J. Zhao , S. Dong , B. He , M. Avdeev , S. Shi , G. Cui , L. Chen , Adv. Mater. 2020, 32, 1907526. 10.1002/adma.201907526 32080916

[7] Q. Wang , M. Zhang , C. Zhou , Y. Chen , J. Phys. Chem. C 2018, 122, 16649.

[8] Z. Jian , C. Yuan , W. Han , X. Lu , L. Gu , X. Xi , Y.‐S. Hu , H. Li , W. Chen , D. Chen , Y. Ikuhara , L. Chen , Adv. Funct. Mater. 2014, 24, 4265.

[9] a) S. Y. Lim , H. Kim , R. A. Shakoor , Y. Jung , J. W. Choi , J. Electrochem. Soc. 2012, 159, A1393;

[10] F. Lalère , V. Seznec , M. Courty , R. David , J. N. Chotard , C. Masquelier , J. Mater. Chem. A 2015, 3, 16198.

[11] Y. C. Liu , N. Zhang , F. F. Wang , X. B. Liu , L. F. Jiao , L. Z. Fan , Adv. Funct. Mater. 2018, 28, 1801917.

[12] F. Y. Xiong , Q. Y. An , L. X. Xia , Y. Zhao , L. Q. Mai , H. Z. Tao , Y. Z. Yue , Nano Energy 2019, 57, 608.

[13] Y. Yu , C.‐H. Chen , Y. Shi , Adv. Mater. 2009, 21, 3541.

[14] a) S. Li , P. Ge , C. Zhang , W. Sun , H. Hou , X. Ji , J. Power Sources 2017, 366, 249;

[15] a) H. Li , X. Yu , Y. Bai , F. Wu , C. Wu , L.‐Y. Liu , X.‐Q. Yang , J. Mater. Chem. A 2015, 3, 9578;

[16] Z. Jian , W. Han , X. Lu , H. Yang , Y.‐S. Hu , J. Zhou , Z. Zhou , J. Li , W. Chen , D. Chen , L. Chen , Adv. Energy Mater. 2013, 3, 156.

[17] C. Zhu , X. Mu , P. A. van Aken , J. Maier , Y. Yu , Adv. Energy Mater. 2015, 5, 1401170.

[18] G. Manohar , K. M. Pandey , S. R. Maity , Ceram. Int. 2021, 47, 32610.

[19] B. Pei , Z. Jiang , W. Zhang , Z. Yang , A. Manthiram , J. Power Sources 2013, 239, 475.

[20] X. Cao , A. Pan , B. Yin , G. Fang , Y. Wang , X. Kong , T. Zhu , J. Zhou , G. Cao , S. Liang , Nano Energy 2019, 60, 312.

[21] B. Zhang , H. Chen , H. Tong , X. Wang , J. Zheng , W. Yu , J. Zhang , J. Li , W. Zhang , J. Alloys Compd. 2017, 728, 976.

[22] Y. Yin , C. Pei , X. Liao , F. Xiong , W. Yang , B. Xiao , Y. Zhao , Z. Ren , L. Xu , Q. J. C. An , ChemSusChem 2021, 14, 2984. 34050630 10.1002/cssc.202100880

[23] Z. Wei , D. Wang , X. Yang , C. Wang , G. Chen , F. Du , Adv. Mater. Interfaces 2018, 5, 1800639.

[24] H. Xiong , M. D. Slater , M. Balasubramanian , C. S. Johnson , T. Rajh , J. Phys. Chem. Lett. 2011, 2, 2560.

[25] E. R. Mucciolo , C. H. Lewenkopf , J. Phys.: Condens. Matter 2010, 22, 273201. 21399249 10.1088/0953-8984/22/27/273201

[26] P. Simon , Y. Gogotsi , B. Dunn , Science 2014, 343, 1210. 24626920 10.1126/science.1249625

[27] Y. Jiang , J. Liu , Energy Environ. Mater. 2019, 2, 30.

[28] T. Brezesinski , J. Wang , S. H. Tolbert , B. Dunn , Nat. Mater. 2010, 9, 146. 20062048 10.1038/nmat2612

[29] a) V. Augustyn , J. Come , M. A. Lowe , J. W. Kim , P.‐L. Taberna , S. H. Tolbert , H. D. Abruña , P. Simon , B. Dunn , Nat. Mater. 2013, 12, 518; 23584143 10.1038/nmat3601

[30] D. W. Hatchett , J. Am. Chem. Soc. 2010, 132, 9220.

[31] P. N. Didwal , R. Verma , C.‐W. Min , C.‐J. Park , J. Power Sources 2019, 413, 1.

[32] A. Van der Ven , J. Bhattacharya , A. A. Belak , Acc. Chem. Res. 2013, 46, 1216. 22584006 10.1021/ar200329r

[33] a) E. Uchaker , Y. Z. Zheng , S. Li , S. L. Candelaria , S. Hu , G. Z. Cao , J. Mater. Chem. A 2014, 2, 18208;

[34] A. Andersson , J. Thomas , J. Power Sources 2001, 97, 498.

