# Supplementary material for: Crystallinity Tuning of Na3V2(PO4)3: Unlocking Sodium Storage Capacity and Inducing Pseudocapacitance Behavior
Source: Adv Sci (Weinh). 2022 Dec 11;10(4):2203552. doi: 10.1002/advs.202203552 (PMC9896047; doi:10.1002/advs.202203552)
Supplement: Supplementary file 1 — Supporting Information [file ADVS-10-2203552-s001.pdf]

Supporting Information

Crystallinity Tuning of $Na_3V_2(PO_4)_3$: Unlocking Sodium Storage Capacity and Inducing Pseudocapacitance Behavior

Hongyang Ma[a,b], Bangchuan Zhao[a,*], Jin Bai[a,*], Peiyao Wang[a,b], Wanyun Li[a,b], Yunjie Mao[a,b], Xiaoguang Zhu[a], Zhigao Sheng[c], Xuebin Zhu[a], and Yuping Sun[a,c]

[a]*Key Laboratory of Materials Physics, Institute of Solid State Physics, HFIPS, Chinese Academy of Sciences, Hefei 230031, People's Republic of China*
[b]*University of Science and Technology of China, Hefei 230026, People's Republic of China*
[c]*High Magnetic Field Laboratory, HFIPS, Chinese Academy of Sciences, Hefei 230031, People's Republic of China*

**Corresponding author.*
E-mail address: jbai@issp.ac.cn (J. Bai); bchzhao@issp.ac.cn (B.Z. Zhao)

Experimental Section

Preparation of carbon foam

The carbon foam (CF) used in this work was obtained by annealing the melamine sponge in argon. At first, the melamine sponge was cut into several pieces with the size of about 5×3×0.5 cm³. The melamine sponge pieces were annealed at 800 °C under an argon atmosphere for 2 h in a tube furnace and then converted to carbon foam. Finally, the obtained CF pieces was punched into disks with a diameter of 12 mm and used as the substrate in the following, the average mass of CF disks is 1-1.5 mg.

Preparation of NVP-E700, NVP-E600 and NVP-S700 materials:

The NVP-E700 sample was synthesized by ESD method. Firstly, 0.8 mmol critic acid, 0.6 mmol NaH_2PO_4, 0.4 mmol NH_4VO_3 and 0.1 mmol glucose were dispersed in 1,2-propanediol solvents (1,2-propanediol: water = 9:1 in volume ratio) by stirring the solvents overnight. Then, the solution was poured into a 20 ml syringe with a 1.2 mm diameter nozzle. In the ESD process, the distance between the substrate and the nozzle was fixed at about 3 cm and the CF substrate was preheated to 240 °C. The voltage applied in the ESD process is 8 kV. The deposited films were annealed at 700 °C for 8 h in a tube furnace under an argon atmosphere and then the NVP-E700 samples can be obtained. The NVP-E600 samples were gained by a similar method except the annealing temperature was settled at 600 °C. For comparison, NVP-S700 sample was synthesized by a conventional sol-gel method. Firstly, 4 mmol critic acid, 3 mmol NaH_2PO_4, 2 mmol NH_4VO_3 and 0.5 mmol glucose were dispersed in 20 ml deionized water, with vigorous magnetic stirring at 80 °C overnight. After water was evaporated from the solution, a black gel was formed. The resulting gel was dried overnight at 120 °C in an oven, followed by grounding and annealing at 700 °C for 8 h in a tube furnace under an argon atmosphere. Then, the black NVP-S700 powder

material was obtained.

Structural and Electrochemical Characterization:

X-ray diffraction (XRD) measurements were performed using a Panalytical X'Pert diffractometer with Cu Kα radiation in a 2θ range from 10 to 80° to characterize the structure of the materials. Their morphologies were observed with field emission scanning electron microscopy (FE-SEM, SU 8020, HITACHI), transmission electron microscope (TEM, Tecnai G2 F20) together with a high-resolution transmission electron microscope (HRTEM) equipment. X-ray photoelectron spectra (XPS) was performed by a Thermo Scientific ESCALAB 250 with Al Kα radiation as the excitation source. Raman spectra were analyzed using a Raman spectroscopy (Renishaw inVia Reflex) with 514.5 nm laser excitation. The Fourier transform infrared spectra (FT-IR) were measured by an FT-IR spectrometer (Thermo Nicolet NEXUS) in the wavenumber of 500-2300 cm^{-1}. Thermogravimetric (TG) analysis was performed on a pyres 1 Perkin Elmer TG thermal analyzer in a temperature range of 50-600 °C with a heating rate of 10 °C min^{-1}. Inductively coupled plasma optical emission spectra (ICP-OES) was performed by a Horiba Ultima Expert.

The electrochemical behavior of the studied materials was evaluated using CR2032 coin cells. The half-cells assembled in a pure argon filled glove box using metallic sodium foil as the counter electrode and glass micro-fiber as the separator. The NVP-S700 cathodes were admixed by the active materials, acetylene black and polyvinylidene fluoride (PVDF) in a weight ratio of 7:2:1, with N-methylpyrrolidone (NMP) as the solvent. The evenly mixed slurry was pasted on Al foil and then dried at 110 °C for 12 h in a vacuum oven. The NVP-E700 and NVP-E600 samples were used as electrodes directly. The loading mass of the electrode is 0.8-1.4 mg. The electrolyte was made of 1 M $NaClO_4$ in ethylene carbonate (EC) and propylene carbonate (PC) (1:1 by volume) with 5% fluoroethylene carbonate (FEC) as the additive. Galvanostatic charge/discharge, rate performance and long cycling capability measurements were performed in the voltage range of 2 to 4.5 V using a Land battery testing system (CT2001A). The cyclic voltammetry (CV) and electrochemical impedance spectroscopy (EIS) tests of the cells were carried out on a CHI660E electrochemical workstation. CV measurements were tested in a voltage range from 2 to 4.5 V. EIS measurements were carried out in the frequency range from 0.01 Hz to 100 kHz, and the amplitude of alternating current was 5 mV.

Supporting figures and tables

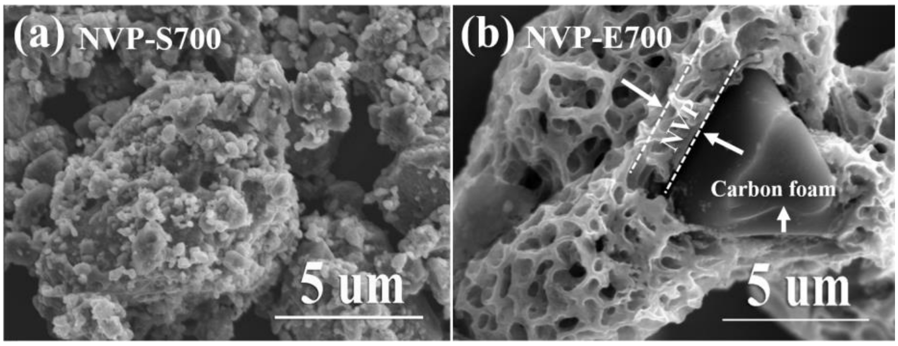

Figure S1. SEM image of (a) the NVP-S700 sample and (b) the cross section of the NVP-E700

sample.

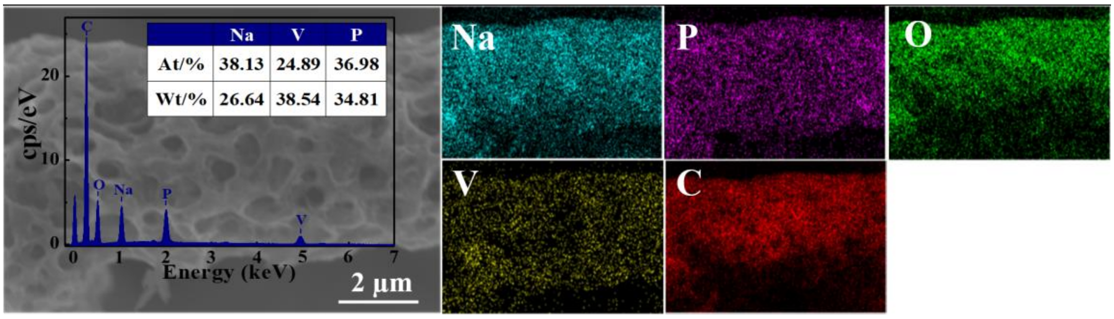

Figure S2. The element mapping images for Na, V, P, O and C elements in the NVP-E700 sample.

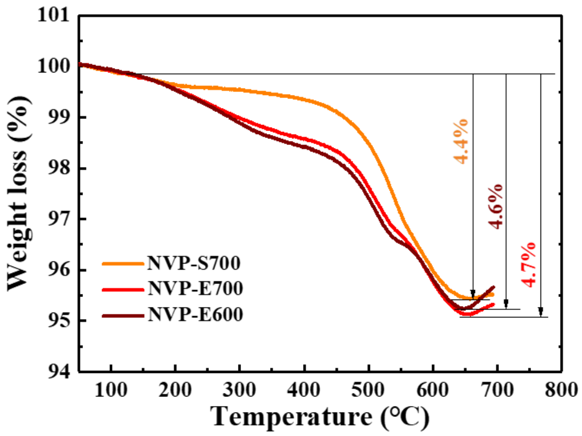

Figure S3. TG plots of the NVP-S700, NVP-E700 and NVP-E600 samples.

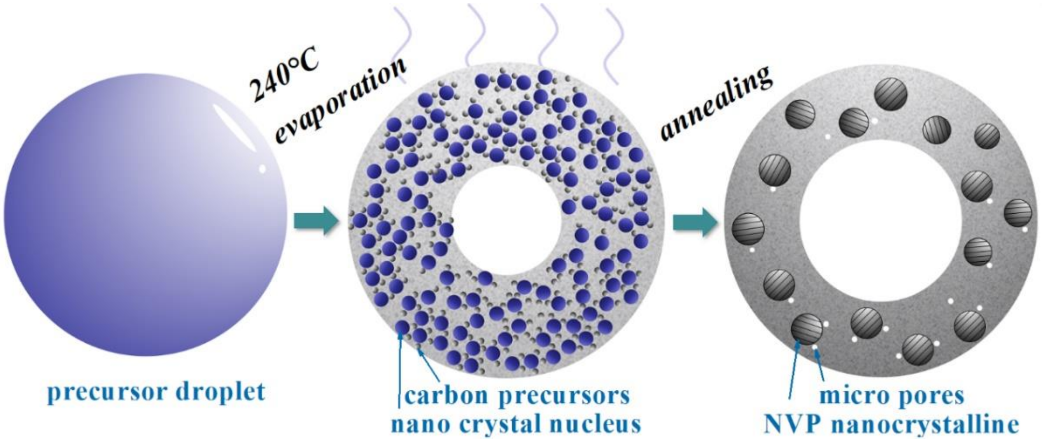

Figure S4. Schematic illustration of the formation mechanism of amorphous and nanocrystalline NVP phase material.

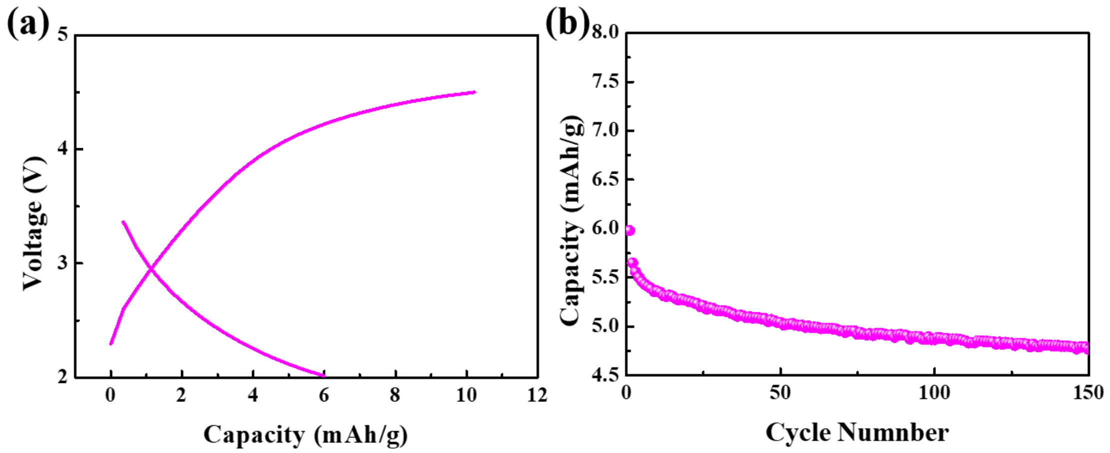

Figure S5. (a) The first cycle galvanostatic charge/discharge profiles and (b) cycle performance of the carbon foam substrate at 0.2 C.

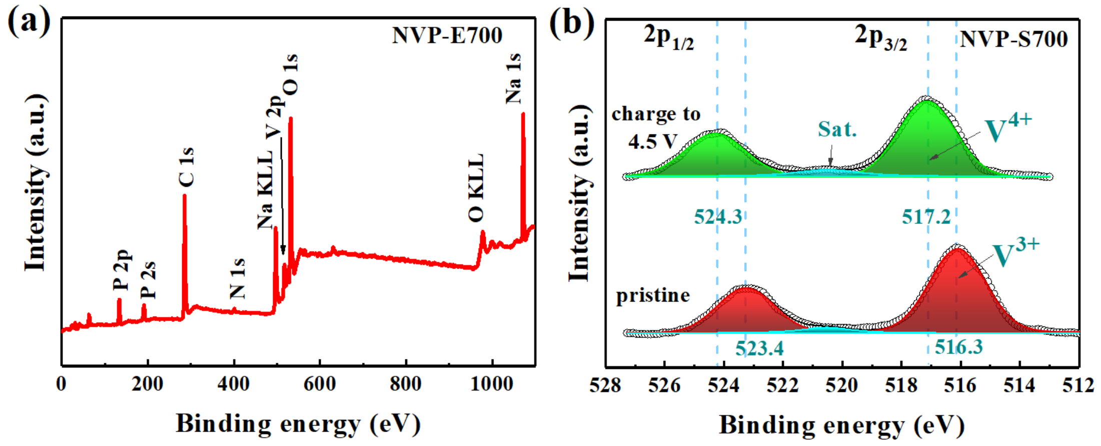

Figure S6. (a) The XPS spectra in a wide-range scanning of the NVP-E700 sample and (b) High-resolution XPS spectra of V for the NVP-S700 sample before and after charged to 4.5V.

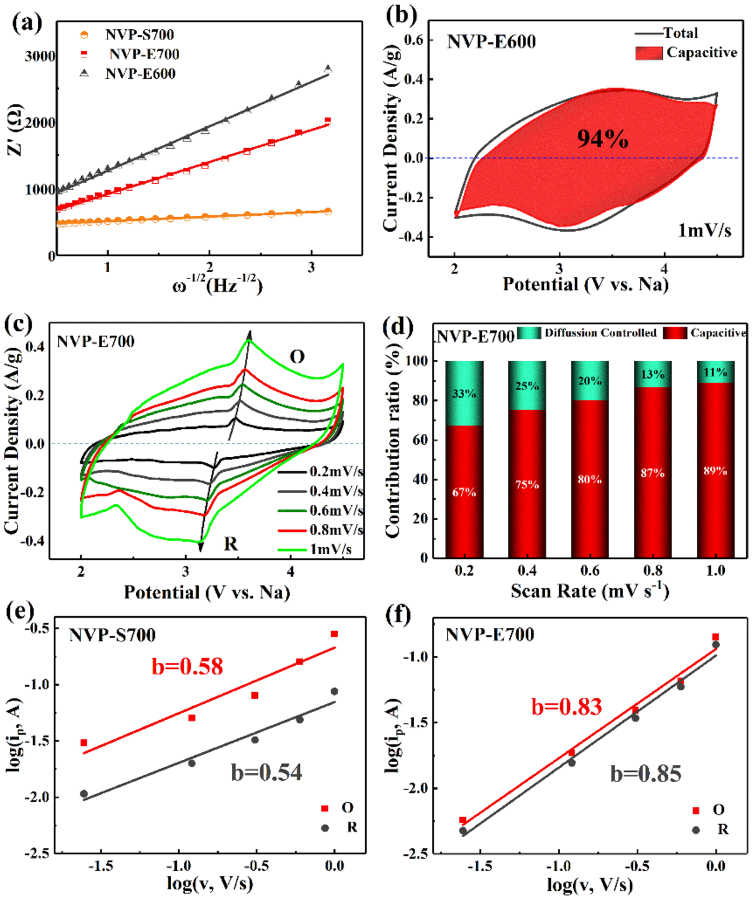

Figure S7. (a) The fitting lines between Z' and $\omega^{-1/2}$ for the NVP-S700 electrodes. (b) Overall capacity with the pseudocapacitive fraction (shaded region) at 1 mV s^{-1} for the NVP-E600 electrode. (c) The CV curves and (d) pseudocapacitive contribution ratios to charge storage at different scan rates from 0.2 to 1 mV•s^{-1} for the NVP-E700 electrode. The log (i_p) vs log (v) plots of (e) NVP-S700 and (f) NVP-E700 electrode.

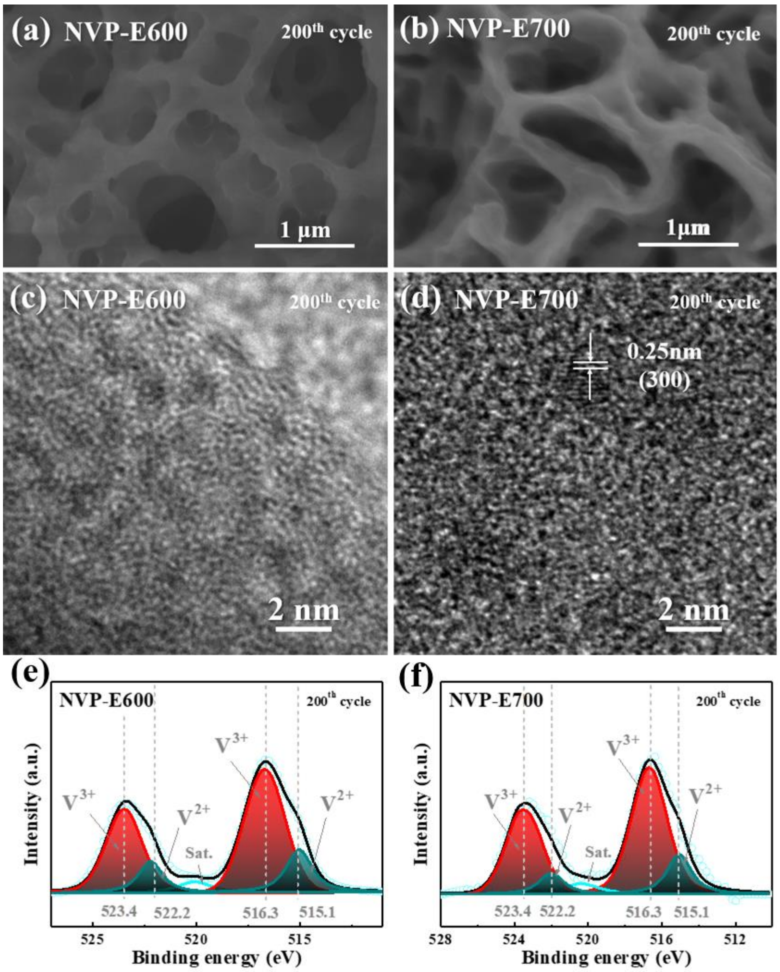

Figure S8. SEM, HRTEM and XPS spectra of V (2p) of (a,c,e) NVP-E600 electrode and (b,d,f) NVP-E700 electrode after 200 cycles.

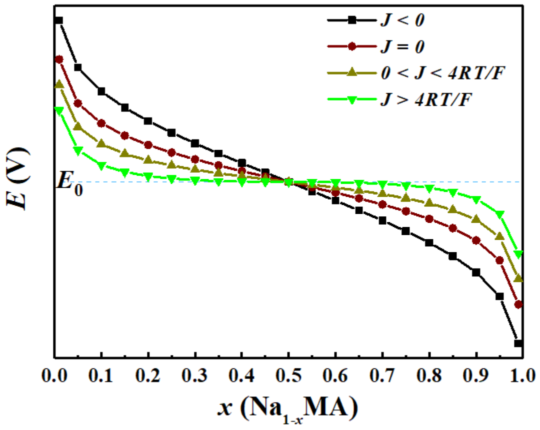

Figure S9. The relationship between E and x at different J value.

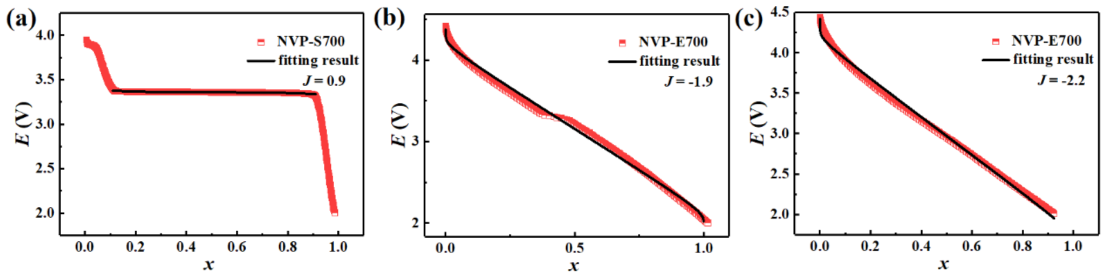

Figure S10. The fitting results of the voltage curves for (a) NVP-S700, (b) NVP-E700 and (c) NVP-E600.

Table S1. Atomic ratio of NVP-S700, NVP-E700 and NVP-E600 samples obtained by ICP-OES test.

Sample	Na (At%)	V (At%)	P (At%)
NVP-S700	37.48	24.99	37.62
NVP-E700	37.72	24.92	37.36
NVP-E600	37.28	25.01	37.71

Table S2. Calculated charge-transfer resistance (R_{ct}) and Na^+ ion diffusion coefficient (D_{Na^+}) for NVP-S700, NVP-E700 and NVP-E600 samples.

Sample	R_{ct} (Ω)	D_{Na^+} ($cm^2 s^{-1}$)
NVP-S700	428	9.77×10^{-14}
NVP-E700	712	2.01×10^{-15}
NVP-E600	827	1.05×10^{-15}
